# Comparing very low birth weight versus very low gestation cohort methods for outcome analysis of high risk preterm infants

**DOI:** 10.1186/s12887-017-0921-x

**Published:** 2017-07-14

**Authors:** Louise IM Koller-Smith, Prakesh S. Shah, Xiang Y. Ye, Gunnar Sjörs, Yueping A. Wang, Sharon S. W. Chow, Brian A. Darlow, Shoo K. Lee, Stellan Håkanson, Kei Lui, on behalf of the Australian and New Zealand Neonatal Network, Peter Marshall, Paul Craven, Karen Simmer, Jacqueline Stack, David Knight, Andrew Watkins, Andrew Ramsden, Kenneth Tan, Kaye Bawden, Lyn Downe, Vjay Singde, Michael Stewart, Andrew Berry, Rod Hunt, Charles Kilburn, Peter Dargaville, Kei Lui, Mary Paradisis, Nick Evans, Shelley Reid, David Cartwright, Carl Kuschel, Lex Doyle, Andrew Numa, Zsuzsoka Kecskes, Nadia Badawi, Guan Koh, Steven Resnick, Mark Tracy, William Tarnow-Mordi, Chad Andersen, Nicola Austin, Brian Darlow, Roland Broadbent, Jenny Corban, Lindsay Mildenhall, Malcolm Battin, David Bourchier, Vaughan Richardson, Ross Haslam, Georgina Chambers, Victor Samual Rajadurai, Canadian Neonatal Network, Shoo K. Lee, Prakesh S. Shah, Andrzej Kajetanowicz, Anne Synnes, Nicole Rouvinez-Bouali, Bruno Piedboeuf, Valerie Bertelle, Barbara Bulleid, Wendy Yee, Sandesh Shivananda, Kyong-Soon Lee, Mary Seshia, Keith Barrington, Francine Lefebvre, Douglas McMillan, Wayne Andrews, Lajos Kovacs, Kimberly Dow, Orlando da Silva, Patricia Riley, Prakeshkumar Shah, Abraham Peliowski, Khalid Aziz, Zenon Cieslak, Zarin Kalapesi, Koravangattu Sankaran, Daniel Faucher, Ruben Alvaro, Roderick Canning, Cecil Ojah, Luis Monterrosa, Michael Dunn, Todd Sorokan, Adele Harrison, Chuks Nwaesei, Mohammed Adie, Swedish Neonatal Quality Register, Stellan Håkansson, Gunnar Sjörs, Niklas Segerdah, Tarek Morad, Stefan Morén, Åke Stenberg, Christer Simonsson, Lennart Stigsson, Jens Ladekjaer Christensen, Lars Åmasn, Fredrik Ingemanson, Laura Österdal, Karl-Gustav Ellström, Thomas Abrahamsson, Ingela Heimdahl, Tomas Hägg, Anna Hedlund, Ellen Elisabeth Lund, Björn Westrup, Ihsan Sarman, Anna Stakkestad Jobe, Magnus Fredsriksson, Anders Palm, Birger Malmström, Eva Lindberg, Owe Ljungdahl, Kerstin Eriksson

**Affiliations:** 1 Faculty of Health Science, University of Technology Sydney, Sydney, NSW Australia; 2 Department of Pediatrics, Mount Sinai Hospital and University of Toronto, Toronto, ON Canada; 3 Maternal Infant Care Research Centre, Mount Sinai Hospital, Toronto, ON Canada; 4 Uppsala University, Uppsala, Sweden; 5 Department of Paediatrics, University of Otago, Christchurch, New Zealand; 6 Umeå University Hospital, Umeå, Sweden; 7 Department of Newborn Care, Royal Hospital for Women, Barker St, Sydney, NSW 2031 Australia

**Keywords:** Outcome, Intensive care, Neonatal, Infant, Premature, Very low birth weight, Small for gestational age, Benchmarking

## Abstract

**Background:**

Compared to very low gestational age (<32 weeks, VLGA) cohorts, very low birth weight (<1500 g; VLBW) cohorts are more prone to selection bias toward small-for-gestational age (SGA) infants, which may impact upon the validity of data for benchmarking purposes.

**Method:**

Data from all VLGA or VLBW infants admitted in the 3 Networks between 2008 and 2011 were used. Two-thirds of each network cohort was randomly selected to develop prediction models for mortality and composite adverse outcome (CAO: mortality or cerebral injuries, chronic lung disease, severe retinopathy or necrotizing enterocolitis) and the remaining for internal validation. Areas under the ROC curves (AUC) of the models were compared.

**Results:**

VLBW cohort (24,335 infants) had twice more SGA infants (20.4% vs. 9.3%) than the VLGA cohort (29,180 infants) and had a higher rate of CAO (36.5% vs. 32.6%). The two models had equal prediction power for mortality and CAO (AUC 0.83), and similarly for all other cross-cohort validations (AUC 0.81–0.85). Neither model performed well for the extremes of birth weight for gestation (<1500 g and ≥32 weeks, AUC 0.50–0.65; ≥1500 g and <32 weeks, AUC 0.60–0.62).

**Conclusion:**

There was no difference in prediction power for adverse outcome between cohorting VLGA or VLBW despite substantial bias in SGA population. Either cohorting practises are suitable for international benchmarking.

**Electronic supplementary material:**

The online version of this article (doi:10.1186/s12887-017-0921-x) contains supplementary material, which is available to authorized users.

## Background

Very premature and very low birth weight (VLBW) infants are at high risk of mortality and morbidities. Effective outcome prediction and benchmarking, for parental counseling, quality improvement and informing the wider community, have their foundation in the outcome statistics of infant cohorts [1]. There are two established methods of cohorting high-risk infants, by birth weight (for example, VLBW, <1500 g) or by gestational age (for example, very low gestational age [VLGA], <32 weeks), with the relative advantages of each yet to be determined. There has been increasing acceptance of gestational age (GA) based cohorting in recent literature [2–5], following studies such as that by Arnold et al. in 1991 [6] and Blair et al. in 1996 [6, 7], which raised concerns that VLBW cohorts may be inherently biased.

Birth weight (BW) is dependent on two separate influences; GA at birth and fetal growth rate [8]. It follows that a VLBW cohort may contain infants at any point along a spectrum from very preterm and sized appropriately for their GA (AGA) to small for gestational age (SGA). There is an inherent selection bias toward SGA infants in VLBW cohorts, which becomes more pronounced at higher gestations, as the birth weights of AGA infants become greater than 1500 g [6, 7, 9]. This disproportionate SGA percentage is exemplified in published studies that have used VLBW cohorts, wherein 19% to 40% of infants were SGA [10–14]. A skewing of risk toward poorer outcome would be expected even in multivariate analyses because high-risk SGA infants lack an equivalent AGA control for adjustment within the cohort. In comparison, GA is independent of BW and fetal growth rate [7], and hence fetal growth and BW for GA show a normal distribution in VLGA cohorts [6]. SGA proportion, by definition, will remain close to 10% across all published cohorts, where SGA percentage ranged from 9.2–12% [15, 16].

Meaningful international examination of neonatal outcomes is currently limited by the variations in reporting between nations [17], as direct comparison of neonatal outcomes through benchmarking requires prior standardization of the infant cohorting method used for data collection and reporting. The World Health Organization changed its standard cohorting practice to GA-based in 1961 [18], but some studies and analyses persist in the use of BW criteria [1, 19–21].

The overall aim of this study was to evaluate and compare the predictive power of prediction models developed using VLGA and VLBW-based cohorts. It was hypothesized that predictive power of the VLGA-based models would be significantly better than that of the VLBW-based models across all networks because it would reduce the selection bias introduced by the disproportionately high number of SGA infants.

## Methods

De-identified clinical data were obtained from the Australian and New Zealand Neonatal Network (ANZNN), Canadian Neonatal Network (CNN) and Swedish Neonatal Quality Register (SNQ) for all infants born either at <32 weeks gestational age or with birth weights <1500 g, who were admitted to participating NICUs in between January 2008 and December 2011. Networks were selected because of their intrinsic similarity, with comparable demographics and healthcare systems. All three networks have the registration criteria for data collection if admitted infants are either <32 weeks or <1500 g. Infants were also excluded if they were moribund (died within the first day of admission without being offered mechanical ventilation or intensive care) or had major congenital anomalies.

The parameters for data collection in each of the network databases were compared. Definitions of outcomes and variables to be analyzed were standardized by consensus a priori. National preterm BW percentiles were examined for each network, and found to be very similar in Australia [22] and Canada [23], however in-utero growth charts were used in the SNQ [24], and therefore the Swedish percentiles were not comparable. For this reason, Canadian BW percentile charts were applied to all infants to define SGA and BW z score.

For the study period, ANZNN data comprised all 29 tertiary hospitals in Australia/New Zealand; CNN comprised 28 of 30 tertiary hospitals in Canada; and SNQ all 25 hospitals with neonatal units in 6 of the 7 health care regions of Sweden. Study data were available through the iNeo (International Network for Evaluating Outcomes in Neonates) project housed at Mother-Infant Care Research Center, Mount Sinai Hospital, University of Toronto, Canada.

The primary outcome studied was in-hospital mortality. The secondary outcome was composite adverse outcome (CAO), defined as in-hospital mortality or a pre-discharge diagnosis of any major neonatal morbidities of chronic lung disease (CLD), serious neurological injuries (SNI) including intraventricular hemorrhage grade III or IV [25] or periventricular leukomalacia, severe retinopathy of prematurity stage 3 or more (ROP) [26] and radiologically or pathologically proven necrotizing enterocolitis (NEC) [27], Consensus outcome definitions are provided in Additional file 1 **:** Table S1. Nosocomial infection was not included in CAO but was included in descriptive analyses, as rate of NI may be used as a marker of patient safety and healthcare effectiveness and outcomes, and hence has relevance for comparison between international cohorts [28].

Data from all networks were amalgamated and formed into two overlapping cohorts of infants less than 32 weeks (VLGA) and/or less than 1500 g (VLBW). Originating network was added as a covariate for subsequent analysis. Of the two overlapping VLBW and VLGA cohorts, two-thirds (balanced for network) were randomly selected, using a split sample method [29], to form the derivation samples for development of two prediction models. The remaining one-third of infants from each cohort formed the internal validation samples, for assessment of predictive power on independent samples.

Prediction models were developed for mortality and CAO by multivariable logistic regression with backwards procedures using exclusion criteria of 0.05, according to methodology validated in previous population studies [30, 31]. The interaction of BW z-score and GA was also included as a covariate in multivariable analysis to adjust for the varying confounding effects of growth status and maturity.

Analysis for prediction power was conducted for each model on both the VLGA and the VLBW validation samples, which consisted of the VLBW and VLGA validation samples, and two mutually exclusive “extreme” subcomponents of infants <1500 g but ≥32 weeks, and infants <32 weeks but ≥1500 g. Prediction power was assessed using area under the Receiver Operating Characteristic (ROC) curve [32–34]. An AUC of >0.80 is generally accepted as excellent prediction [35]. AUC of each prediction was compared. Goodness-of-fit was determined by use of the Hosmer-Lemeshow test [13] to test for systematic over or underestimation of outcomes by the model [36]. Data management and analyses were performed using SAS 9.3 [37] and R 2.10.15 [38]. A two-sided significance level of 0.05 has been used without adjustment for multiple comparisons.

ANZNN data collection, access and use of de-identified data for audit and research was approved by all relevant institutional research ethics committees of each NICU hospital (see list of hospitals in Acknowledgement) in Australia, and by the New Zealand Multi-regional Ethics Committee for all the New Zealand hospitals listed. For the CNN and SNQ, de-identified data collection was approved at each site by either an institutional ethics board or quality improvement committee of the hospitals listed. All participating networks have obtained ethics/regulatory approval or the equivalent from their local granting agencies to allow for de-identified data to be collated. De-identified ANZNN, CNN and SNQ data were amalgamated at the iNeo collaboration centre where analysis occurred. Approval for this project was obtained from the South Eastern Sydney Local Health District Human Research Ethics Committee and approval for data transfer was obtained from all three networks executive committees. The ethics committees waived the requirement for the consent. Data from all networks were amalgamated and used for this study.

The Coordinating Centre has been granted Research Ethics Board approval for the development, compilation, and hosting of the dataset, and all 3 networks have signed data transfer agreements with the Coordinating Centre. Privacy and confidentiality of patient and unit-related data will be of prime importance to the iNeo collaboration, and data collection, handling, and transfer will be performed in accordance with the Canadian Privacy Commissioner’s guidelines, the Personal Information Protection and Electronic Documents Act, and any other local rules and regulations. No data identifiable at the patient level will be collected or transmitted, and only aggregate data will be reported. For all stages of the project, participating units will be assigned a code by their own network prior to data transfer into the iNeo dataset so that units remain anonymous within the iNeo collaborative. Following data analysis, findings will be disseminated within networks by their own network coordination team and not by the iNeo central team.

Following completion of the study in 2017, the data will be kept at the iNeo Coordinating Centre for a further 2 years before being returned to the originating networks unless otherwise agreed by the member networks.

## Results

**Fig. 1:**
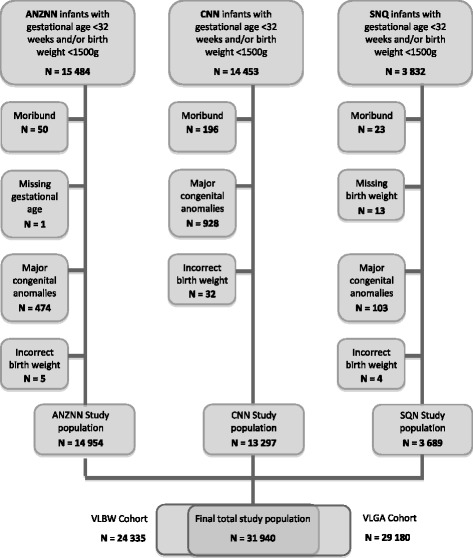
Flow Chart of Study Cohort: derivation of the study infants from each neonatal network is summarised

The derivation of the study cohort is detailed in Fig. 1. The final study population contained 31,940 infants; 14,954 infants from the ANZNN (46.8%), 13,297 (41.6%) from the CNN and 3689 (11.5%) from the SNQ. The VLBW cohort was made up of 24,335 infants (76.2% of study population) and the VLGA cohort of 29,180 infants (91.4% of study population).

The majority of infant characteristics were similar between the VLGA and VLBW cohorts (see Table 1), with the expected exception of SGA percentage, which was more than double in the VLBW cohort (20.4%) compared to the VLGA cohort (9.3%). For the VLGA cohort, mean GA was marginally higher for ANZNN infants (28.5 weeks) than for CNN or SNQ infants (28.3 weeks). Antenatal steroid use was significantly lower in the CNN than ANZNN and SNQ. Significantly fewer infants required exogenous surfactant in the ANZNN than CNN or SNQ. Similar disparities between networks were seen in the VLBW cohort.

**Table 1:** Comparisons of infant and perinatal characteristics and neonatal outcomes among networks (ANZNN, CNN, SNQ) for very low gestational age cohort and very low birth weight cohort derived from network admissions 2008–2011

	Very low gestational age				Very low birth weight			
Networks	ANZNN	CNN	SNQ	*P*-value	ANZNN	CNN	SNQ	*P*-value
Total number	13,586	12,167	3427		11,307	10,327	2701	
Infant and Perinatal Characteristics								
Gestational age, mean(SD)	28.5(2.2)	28.3(2.2)	28.3(2.4)	<0.0001	28.3(2.6)	28.2(2.6)	27.9(2.7)	<0.0001
Birth weight, mean(SD)	1232 396)	1209 (387)	1234 (424)	<0.0001	1082 (274)	1068(267)	1055 (291)	<0.0001
Male sex	7345 (54.1)	6617 (54.5)	1838 (53.6)	0.6619	5762 (51.0)	5290(51.3)	1372 (50.8)	0.8571
Small for gestational age	1249 (9.2)	1133 (9.3)	317 (9.3)	0.9515	2369 (21.0)	2082(20.2)	521 (19.3)	0.0999
Singleton	9532 (70.2)	8417 (69.3)	2441 (71.3)	0.0478	7901 (69.9)	7099(68.8)	1930 (71.5)	0.016
Surfactant required	7336 (54.1)	7179 (59.0)	1941 (56.8)	<0.0001	6442 (57.1)	6233(60.4)	1649 (61.3)	<0.0001
Antenatal steroid use	12,902 (95.1)	10,156 (86.6)	3278 (95.7)	<0.0001	10,723 (95.0)	8520(85.7)	2581 (95.6)	<0.0001
Caesarean	8478 (62.7)	7117 (59.0)	2086 (61.2)	<0.0001	7311 (65.0)	6565(64.1)	1679 (62.7)	0.0613
Presentation (vertex)	8676 (65.4)	7307 (64.7)	2209 (65.7)	0.3872	7099 (64.2)	6004(63.0)	1720 (65.3)	0.0353
Maternal age, mean(SD)	29.8 (6.4)	30.5 (5.9)	29.9 (6.5)	<0.0001	29.7 (6.5)	30.5(6.0)	29.8 (6.6)	<0.0001
Neonatal Outcomes								
Mortality	987 (7.3)	986 (8.1)	265 (7.7)	0.0407	948 (8.4)	945(9.2)	252 (9.3)	0.0841
Severe neurological injury	758 (5.6)	1409 (9.4)	220 (6.4)	<0.0001	711 (6.3)	1293(10.1)	202(7.5)	<0.0001
Severe retinopathy of prematurity	496 (3.7)	592 (4.9)	146 (4.3)	<0.0001	487 (4.3)	596(5.8)	144 (5.3)	<0.0001
Necrotising enterocolitis	591 (4.4)	690 (5.7)	143 (4.2)	<0.0001	566 (5.0)	641(6.2)	134 (5.0)	0.0002
Chronic lung disease	2381 (17.5)	2597 (21.3)	629 (18.4)	<0.0001	2233 (19.8)	2472(23.9)	586 (21.7)	<0.0001
Composite adverse outcome	3893 (28.7)	4564 (36.5)	1043 (30.4)	<0.0001	3649 (32.3)	4272(40.3)	962 (35.6)	<0.0001
Nosocomial Infection	2008 (14.8)	2188 (18.0)	484 (14.1)	<0.0001	1869 (16.5)	2069(20.0)	454 (16.8)	<0.0001

N (%) are shown unless specified

Notes: the reported *p*-values were based on chi-square tests for categorical variables, and F tests for continuous variables. Composite adverse outcome is defined as: death or any major morbidities including chronic lung disease, severe neurological injury, necrotising enterocolitis, severe retinopathy. Nosocomial infection is not included

### Neonatal outcomes

In the VLGA cohort (Table 1), mortality rates were similar across networks while CAO was higher in the CNN (36.5%) than the ANZNN (28.7%) and SNQ (30.3%). The same trends were observed in the VLBW cohort. Mortality rates were similar between the VLBW (8.9%) and VLGA (7.7%) cohorts. The greatest disparity was in the higher rates of CLD (21.7% vs. 19.2%) and NI (18.1% vs. 16.0%) in the VLBW cohort. CAO rate was higher (36.5% vs. 32.6%) in the VLBW cohort, beyond the effect of the increased CLD incidence. The neonatal outcomes of all 3 networks stratified by gestation subgroups (22–24, 25–26, 27–28 and 29–31 weeks) and birth weight (<750 g, 750-999 g, 1000-1249 g and 1250–1499 g) subgroups are summarised in Additional file 1: Table 2 (a) and (b).

**Table 2:** Predictive models for mortality developed using data from ANZNN, CNN and SNQ 2008–2011

	(a) Model based on BW cohort			(b) Model based on GA cohort		
Covariates	Estimate	SE	*P*-value	Estimate	SE	*P*-value
Intercept	14.1797	0.4661	<0.0001	12.9809	0.3814	<0.0001
GA (weeks)	−0.6128	0.0182	<0.0001	−0.5629	0.0146	<0.0001
BW z score	1.5657	0.3677	<0.0001	#		
GA x BW z score	−0.067	0.014	<0.0001	#		
Country (Sweden vs CA)	#			#		
Country (Australia vs CA)	#			#		
Gender (male)	0.3499	0.0642	<0.0001	0.2969	0.0616	<.0001
Antenatal steroid use	−0.6825	0.104	<0.0001	−0.5745	0.1008	<.0001
Singleton	#			#		
Cesarean	#			#		
Presentation (Vertex)	−0.1562	0.0639	0.015	−0.2213	0.0611	0.0003
Area under ROC curve	0.8303	0.0058		0.828	0.00585	
Hosmer-Lemeshow test			0.18			0.15

Multiple logistic regression models were applied to obtain the final predictive models using stepwise variable selection procedure with inclusion and exclusion criterion of 0.05; # = excluded by the variable selection procedure

Notes: *BW z score* Birth weight z score, *GA x BW z score* interaction between GA and Birth weight z score, *Estimate* estimated coefficient of the covariate, *SE* standard error

### VLGA and VLBW prediction models for mortality and composite adverse outcome

Mortality prediction models developed using the VLBW and VLGA derivation cohorts are shown in Table 2. AUC was analogous for the VLBW (0.830) and VLGA (0.828) based models, and both models had equally good discriminatory power. The CAO prediction models (Table 3) included similar variables to the mortality prediction models. Both the VLGA and VLBW-based models showed equal discrimination, with an AUC of 0.83.

**Table 3:** Predictive models for composite adverse outcome developed using data from ANZNN, CNN and SNQ 2008–2011

	(a) Model based on BW cohort			(b) Model based on GA cohort		
Covariates	Estimate	SE	*P*-value	Estimate	SE	*P*-value
Intercept	18.66	0.363	<0.0001	17.8	0.285	<0.0001
GA (weeks)	−0.682	0.013	<0.0001	−0.649	0.01	<0.0001
BW z score	1.386	0.272	<0.0001	−0.15	0.022	<0.0001
GA x BW z score	−0.057	0.01	<0.0001	#		
Country (sweden vs CA)	−0.427	0.07	<0.0001	−0.326	0.065	<0.0001
Country (Australia vs CA)	−0.416	0.045	<0.0001	−0.371	0.042	<0.0001
Gender (male)	0.192	0.042	<0.0001	0.257	0.039	<0.0001
Antenatal steroid use	−0.259	0.08	0.0012	−0.237	0.075	0.002
Singleton	#			#		
Cesarean	0.3	0.044	<0.0001	0.241	0.04	<0.0001
Presentation (Vertex)	#			#		
Area under ROC curve	0.837	0.0033		0.835	0.003	
Hosmer-Lemeshow test			0.19			0.373

Multiple logistic regression models were applied to obtain the final predictive models using stepwise variable selection procedure with inclusion and exclusion criterion of 0.05; # = excluded by the variable selection procedure

Notes: *BW z score* Birth weight z score, *GA x BW z score* interaction between GA and Birth weight z score, *Estimate* estimated coefficient of the covariate, *SE* standard error

### Application of prediction models to validation samples

When applied to the validation samples, the predictive power of both the VLBW-based and VLGA- based models remained excellent (AUC 0.81–0.85) for prediction of mortality and CAO. Cross-comparison showed equivalent performance between the VLBW and VLGA-based models for application to both the VLBW and VLGA developmental samples (Additional file 1: Table 3). Statistical significance (*p* < 0.05) was reached between the VLGA and VLBW-based CAO prediction models for the VLBW validation sample. Predictive power of the VLBW and VLGA-based models remained excellent across networks and exhibited a narrow range in AUC of 0.81 to 0.86. This demonstrated the applicability of the developed models to the three included networks. Due to the large sample sizes, statistical significance was shown between the VLGA and VLBW models for some comparisons despite very small differences.

### Application of prediction models to extreme subsets

There are two mutually exclusive subset of the cohorts: 2759 infants in the VLBW cohort whose gestation was 32 weeks or above and 7603 infants in the VLGA cohort whose birthweight was 1500 g or more. Predictive power decreased when the models were applied to these two extreme subset of the VLBW and VLGA cohorts (AUC 0.50–0.62) (Table 4). Neither model demonstrated consistently better performance for the prediction of mortality or CAO in either of the extreme subsets. In the VLBW and ≥32 week extreme subsets, most (2273/2759, 82.4%) were SGA, and conversely in the extreme VLGA and ≥1500 g subset a smaller proportion (713/7603, 9.3%) were SGA and a considerable number of infants (1300/7603, 17.1%) were large for gestation age (LGA). Both extreme subsets had consistently lower crude morbidity and composite adverse outcome rates than those of the total cohorts (Additional file 1: Table 4).

**Table 4:** Cross comparison of predictive power of the very low birth weight (VLBW) and very low gestational age (VLGA) based prediction models for application to the total extreme subsets of the VLGA and VLBW verification cohorts

Extreme subsets	BW <1500 & GA ≥32 wk	GA <32 & BW ≥1500
	*n* = 893	*n* = 2527
Model for mortality based on VLBW cohort	0.605	0.623
Model for mortality based on VLGA cohort	0.504	0.618
Model for CAO based on VLBW cohort	0.649	0.608‡
Model for CAO based on VLGA cohort	0.618	0.622‡

Notes: †: *p* < 0.01,‡: *p* < 0.05; Two figures are significantly different if they share the same symbol. Chi-square test was used for the comparison in predictive power of the two models

## Discussion

This study is the first to systematically assess the comparative predictive power of VLBW and VLGA cohorting methods. Belief in the superiority of gestation-based cohorts has grown amongst many investigators [2, 3, 39–41] in response to suggestion that VLBW cohorts are limited by their innate confounding of growth status and maturity [6–8, 17, 42], but have not been formally validated. In this retrospective population study of 31,940 neonates from Australia/New Zealand, Canada and Sweden, we identified that outcome prediction models derived from VLGA and VLBW cohorts perform equally well for prediction of in-hospital mortality and CAO in these high-risk preterm infants.

As expected, the VLBW study cohort held a disproportionately high number of SGA infants [6–8, 17, 42] in harmony with previous large population studies, which show SGA proportions of 20–39% in VLBW groups [10–13, 43, 44] compared to 8–12% in VLGA groups [15, 16, 45–47].

The expected skewing of risk toward poor outcome in VLBW cohorts was confirmed by the higher rate of CAO in this group (36.5%) compared to the VLGA group (32.6%). The VLBW study cohort had higher rates of NI (18.1% vs. 16.0%) and CLD (21.7% vs. 19.2%) than the VLGA cohort across all networks [48] confirming previous studies that SGA infants have higher risk of CLD [49–53] and NI [50, 52, 54] compared to AGA infants of the same GA. Previous studies have also suggested higher mortality [49–51] and NEC [52] rates in SGA infants, yet inconclusive as to whether SGA groups have excess risk of severe ROP and SNI [48, 50, 52, 54]. The current international study has the largest sample size of any research examining these morbidities and thus has the statistical power to determine small differences in outcome. The smaller than hypothesised outcome difference found between the VLGA and VLBW groups is likely related to improvement of SGA outcomes associated with advances in contemporary clinical practice. The protective effect (negative coefficient) found for vertex presentation for both VLBW and VLGA cohorts suggests other presentations such as breech, transverse or others are associated with a less favourable outcome.

No clinically significant difference in predictive performance was found between the VLGA and VLBW models in this study. The higher SGA percentage within the VLBW cohort did not affect the discrimination power of the VLBW model, suggesting adequate control within the model for the confounding effect present. We propose two explanations for the rejection of our hypothesis. First, in previous VLBW cohort publications, many infants may not have had accurate prenatal gestation assessments, primarily due to substantial limitations in accessibility to early dating ultrasound. In comparison, GA assessment in the three networks of this contemporary study was robust, as all three networks have national healthcare access with nearly universal ultrasound examinations of pregnancies. The accurate GA data in both the VLGA and VLBW cohorts improved the accuracy of the models in this study, compared to expectations from previously published VLBW cohort data. Second, the research methodology of this study allowed for inclusion of non-linear relationships, such as the GA and BW z-score interaction. In the VLBW models, this adjusted for growth status and maturity through a balanced shift in the coefficients for BW z-score and GA as well as the negative coefficient in their interaction being the protective confounding effect of growth status and maturity. The non-inclusion of these covariates in the VLGA models likely reflects that similar adjustment for SGA infants was not needed, as expected in keeping with the consistent 10% SGA. Consequently, the large sample sizes of this study combined with sophisticated modelling allowed development of models able to effectively control for confounding and bias, leading to the null findings.

Comparison of the models’ usefulness for prediction in the two ‘extreme’ subsets of VLGA-not-VLBW and VLBW-not-VLGA tests the scope of application. It was found that the power of all models fell when applied to the <1500 g BW ≥32-week GA infants, who would almost all be moderately or severely SGA. Predictive power also dropped for both the VLGA and VLBW models when used for CAO prediction in the BW ≥1500 g and GA <32-week subset, but remained excellent for mortality prediction. This clearly confirms that both mortality prediction models perform well for an extreme cohort containing no SGA infants [23]. The finding that the CAO prediction model did not perform as well as expected could indicate increased vulnerability of large for GA infants to morbidities. The findings suggest that separate prediction models may need to be developed for infants on the extreme subsets of established cohorts where there is a high proportion of SGA or LGA infants, as standard statistical modelling derived from either VLGA or VLBW may not be appropriate for use.

This study is reliable due to its large sample size of 31,940, and the population based nature of the data [55]. Relative to the size of the samples there were very few missing or incorrect data, attesting to the high quality of the originating databases. The international collaboration allowed validation of study findings across three neonatal networks, and was made more effective by choosing networks with similar databases. Additionally, Canada, Sweden and Australia/New Zealand have high coverage with early dating ultrasound and thus accurate GA data, in contrast to other studies that have combined last menstrual period and ultrasound dating, thus applying GA estimations that differ by up to 3 weeks [8, 56]. Through the examination of both mortality and CAO, this study will be useful as survival at lower GA becomes possible and prediction of survival without major morbidities becomes increasingly vital.

This study was limited to the analysis of variables collected uniformly across all network databases for the complete study period, but the similarity and quality of the network databases included curtailed the effect of this limitation. The observational, retrospective design meant that no causal mechanisms can be imputed..

The conclusion that VLBW cohorts perform as well as VLGA cohorts for prediction of mortality and morbidity will have ramifications at the international and population levels. Comparison of population outcome may now be considered valid regardless of the cohorting method used to obtain data, providing both GA and BW z scores are included in analytic models. This represents a major advancement in international benchmarking. This study also provides evidence to justify the continued use of BW-based cohorting in some nations provided accurate GA data are included. A further corollary of this study is clarification of the literature on VLGA and VLBW neonates. The findings elucidate both the external validity of research based on one cohort for application to the other, and the appropriateness of comparing data or conclusions based on disparately cohorted groups.

Further investigation is warranted into whether the findings of this study can be extrapolated to countries with poorer access to antenatal care, in particular early dating ultrasound, and hence less accurate GA estimation. Moreover, further studies should compare predictive power for longer-term outcomes such as neurodevelopment, where differing SGA proportions would be expected to have greater effect.

## Conclusion

Outcomes of high-risk neonates are commonly reported either by gestational age or by birth weight. Compared to gestation-based cohorts, birth weight cohorts are more prone to selection bias toward small-for-gestational age infants, who are at high risk of adverse outcomes. However, this study found cohorts based on VLBW or VLGA were equally effective when used to generate prediction models for mortality and morbidity across the three national neonatal networks of Australia/New Zealand, Canada and Sweden. Both models had excellent predictive power when applied to VLGA and VLBW groups, illustrating that either model is appropriate for use, provided GA and BW parameters included in the modeling have been collected well. Neither model performed well at the extremes of BW for GA, particularly where it contained a high proportion of SGA or LGA infants. The findings of this study may facilitate comparisons for international benchmarking and subsequent quality improvement, and provides support for continued adherence to BW-based cohorting in appropriately designed population studies.

## Additional files

Additional file 1: Table S1. Consensus definitions of important variables. Definitions of neonatal outcomes with consensus definitions agreed upon by the CNN, ANZNN and SNQ. **Table S2**: Stratified outcomes between networks. (a): Unadjusted perinatal risks, mortality and major neonatal morbidities among SNQ, ANZNN and CNN infants during 2008–2011 by gestational age groups. (b): Unadjusted perinatal risks, mortality and major neonatal morbidities among SNQ, ANZNN and CNN infants during 2008–2011 by birth weight groups. **Table S3**: Cross comparison of predictive power of very low birth weight (VLBW) and very low gestational age (VLGA) based models. **Table S4**: Comparisons of infant and perinatal characteristics and neonatal outcomes among networks (ANZNN, CNN, SNQ) for the 2 extreme components of the very low gestational age cohort and very low birth weight cohort 2008–2011 admissions [25–27, 57–59]. (DOC 174 kb)

## Abbreviations

AGA: Appropriate for gestational age
ANZNN: Australia and New Zealand Neonatal Network
AUC: Area under the curve
BW: Birth weight
CAO: Composite adverse outcome
CLD: Chronic lung disease
CNN: Canadian Neonatal Network
GA: Gestational age
IVH: Intraventricular haemorrhage
NEC: Necrotising enterocolitis
NI: Nosocomial infection
NICU: Neonatal intensive care unit
PVL: Periventricular leukomalacia
ROC: Receiver operating characteristic
ROP: Retinopathy of prematurity
SD: Standard deviation
SGA: Small for gestational age
SNI: Severe neurological injury
SNQ: Swedish Neonatal Quality Register
VLBW: Very low birth weight
VLGA: Very low gestational age
