# Supplementary material for: Comparing very low birth weight versus very low gestation cohort methods for outcome analysis of high risk preterm infants
Source: BMC Pediatr. 2017 Jul 14;17:166. doi: 10.1186/s12887-017-0921-x (PMC5512978; doi:10.1186/s12887-017-0921-x)
Supplement: Supplementary file 1 — Consensus definitions of important variables. Definitions of neonatal outcomes with consensus definitions agreed upon by the CNN, ANZNN and SNQ. Table S2: Stratified outcomes between networks. (a): Unadjusted perinatal risks, mortality and major neonatal morbidities among SNQ, ANZNN and CNN infants during 2008–2011 by gestational age groups. (b): Unadjusted perinatal risks, mortality and major neonatal morbidities among SNQ, ANZNN and CNN infants during 2008–2011 by birth weight groups. Table S3: Cross comparison of predictive power of very low birth weight (VLBW) and very low gestational age (VLGA) based models. Table S4: Comparisons of infant and perinatal characteristics and neonatal outcomes among networks (ANZNN, CNN, SNQ) for the 2 extreme components of the very low gestational age cohort and very low birth weight cohort 2008–2011 admissions [25–27, 57–59]. (DOC 174 kb) [file 12887_2017_921_MOESM1_ESM.doc]

**Additional file 1**

**Table S1:Consensus definitions of important variables. Definitions of neonatal outcomes with consensus definitions agreed upon by the CNN, ANZNN and SNQ**

| **Outcome** | **CNN Definition** | **ANZNN Definition** | **SNQ Definition** | **Consensus Definition** |
| --- | --- | --- | --- | --- |
| **Mortality** | Death before last discharge from NICU | Death before discharge home | Death this care episode | Death prior to discharge |
| **Chronic Lung Disease (CLD)** | Oxygen use at 36 weeks corrected gestational age  (death before 36wks, missing; transfer before 36wks in oxygen, CLD) | Oxygen use at 36 weeks corrected gestational age  (death before 36wks, missing;  transfer before 36wks, reported from receiving hospital) | Oxygen use at 36 weeks (death before 36wks, based on X-ray /autopsy findings) | Oxygen use at 36 weeks corrected gestational age among survivors with available data |
| **Severe Neurological Injury**  **(SNI)** | Intraventricular haemorrhage (IVH) grade III-IV according to Papile , or periventricular leukomalacia (PVL: echogenic diffuse cystic brain lesions in white matter).  Questionable/possible reported as absent. | Worst grade IVH in either hemisphere by imaging or post-mortem in first 10 days of life. Grading by Papile  PVL: echogenicitiy or cystic changes affecting periventricular white matter, measured by ulstrasound at age closest to 6 weeks | IVH: Grade 3-4 according to Papile  PVL: cystic change or echodensity on ultrasound (missing if not investigated after 4 wks) | Intraventricular haemorrhage with ventricular enlargement or persistent parenchymal echogenicity (grade III-IV); or periventricular leukomalacia |
| **Severe Necrotising Enterocolitis**  **(NEC)** | Stage 2 or higher according to Bell’s criteria | Stage 2 or higher according to Bell’s criteria | Stage 2 or higher according to Bell’s criteria | Stage 2 or higher according to Bell’s criteria |
| **Severe Retinopathy of Prematurity**  **(ROP)** | Maximum stage of ROP in left/right eye Stage 3 or above according to International Committee on Retinopathy of Prematurity (ICROP) | Worst stage of ROP seen in either eye is stage 3 or above according to ICROP | Maximum ROP stage during current hospitalization for each eye, according to the stages defined by ICROP. | Stage 3 or higher as defined by the ICROP |

**Table S2: Stratified outcomes between networks. (a): Unadjusted perinatal risks, mortality and major neonatal morbidities among SNQ, ANZNN and CNN infants during 2008-2011 by gestational age groups**

|  |  |  | **Gestational Age at Birth (Weeks), N (%)** | | |
| --- | --- | --- | --- | --- | --- |
| **Outcomes** | **Network** | 22 - 24 | 25 - 26 | 27 - 28 | 29 - 31 |
| **Antenatal Steroid** | **SNQ** | 318 (96.1) | 461 (97.1) | 732 (96.1) | 1767 (95.2) |
|  | **ANZNN** | 843 (95.6) | 2068 (96.9) | 2830 (95.3) | 7161 (94.4) |
|  | **CNN** | 610 (76.6)** | 1754 (88.2)** | 2401 (88.8)** | 5391 (86.4)** |
| **Small for Gestational Age** | **SNQ** | 28 (8.4) | 53 (11.2) | 92 (12.07) | 144 (7.7) |
|  | **ANZNN** | 40 (4.5) | 219 (10.3) | 317 (10.7) | 673 (8.9) |
|  | **CNN** | 38 (4.6)† | 150 (7.3)** | 276 (9.8) | 669 (10.3)* |
| **Composite Adverse Outcome** | **SNQ** | 299 (89.8) | 307 (64.6) | 244 (32.2) | 193 (10.4) |
|  | **ANZNN** | 782 (88.2) | 1389 (65.0) | 991 (33.3) | 731 (9.6) |
|  | **CNN** | 746 (90.4) | 1446 (70.0) * | 1203 (42.8)** | 1045 (16.2)** |
|  | **SNQ** | 130 (39.0) | 69 (14.5) | 35 (4.6) | 31 (1.7) |
| **Mortality** | **ANZNN** | 328 (36.9) | 357 (16.7) | 184 (6.2) | 118 (1.6) |
|  | **CNN** | 360 (43.6)† | 337 (16.3) | 164 (5.8) | 125 (1.9) |
| **Chronic Lung Disease** | **SNQ** | 154 (46.3) | 199 (41.9) | 161 (21.1) | 115 (6.2) |
|  | **ANZNN** | 419 (47.2) | 897 (42.0) | 657 (22.1) | 408 (5.4) |
|  | **CNN** | 329 (39.9)* | 891 (43.2) | 792 (28.2)** | 585 (9.0)* |
| **Severe Neurological Injury** | **SNQ** | 74 (22.2) | 70 (14.7) | 38 (5.0) | 38 (2.1) |
|  | **ANZNN** | 206 (23.2) | 237 (11.1) | 159 (5.4) | 156 (2.1) |
|  | **CNN** | 256(31.0)* | 355(17.2)** | 263(9.4)** | 264(4.1)** |
| **Retinopathy of Prematurity** | **SNQ** | 69 (20.7) | 50 (10.5) | 19 (2.5) | 8 (0.4) |
|  | **ANZNN** | 176 (19.8) | 226 (10.6) | 64 (2.2) | 30 (0.4) |
|  | **CNN** | 185 (22.4) | 291 (14.1)* | 88 (3.1) | 28 (0.4) |
| **Necrotising Enterocolitis** | **SNQ** | 45 (13.5) | 43 (9.1) | 39 (5.1) | 16 (0.9) |
|  | **ANZNN** | 137 (15.5) | 209 (9.8) | 139 (4.7) | 106 (1.4) |
|  | **CNN** | 103 (12.5) | 194 (9.4) | 196 (7.0)** | 197 (3.1)** |
| **Nosocomial Infection** | **SNQ** | 131 (39.3) | 151 (31.8) | 123 (16.1) | 79 (4.3) |
|  | **ANZNN** | 357 (40.3) | 712 (33.3) | 499 (16.8) | 440 (5.8) |
|  | **CNN** | 329 (39.9) | 696 (33.7) | 630 (22.4)** | 533 (8.2)** |
| Notes: ** indicates p<0.001, * p<0.01, and †p<0.05 (for comparison among SNQ, ANZNN and CNN). The p-values were based on the Chi-square test. | | | | | |

**(b): Unadjusted perinatal risks, mortality and major neonatal morbidities among SNQ, ANZNN and CNN infants during 2008-2011 by birth weight groups**

|  |  |  | **Birth Weight (Grams), N (%)** | |  |
| --- | --- | --- | --- | --- | --- |
| **Outcomes** | **Network** | <750 | 750 - 999 | 1000-1249 | 1250 -1499 |
|  | **SNQ** | 479 (96.2) | 595 (95.2) | 679 (95.4) | 828 (95.8) |
| **Antenatal Steroid** | **ANZNN** | 1568 (95.3) | 2559 (95.9) | 2984 (95.5) | 3612 (93.7) |
|  | **CNN** | 1240 (84.9)** | 2240 (88.3)** | 2489 (86.8)** | 2551 (82.9)** |
| **Small for Gestational Age** | **SNQ** | 156 (31.3) | 110 (17.6) | 106 (14.9) | 149 (17.3) |
|  | **ANZNN** | 561 (34.0) | 485 (18.1) | 570 (18.2) | 753 (19.5) |
|  | **CNN** | 482 (32.1) | 469 (17.9) | 539 (18.1) | 592 (18.4) |
| **Composite Adverse Outcome** | **SNQ** | 376 (75.34) | 306 (48.9) | 182 (25.6) | 98 (11.3) |
|  | **ANZNN** | 1114 (67.6) | 1296 (48.5) | 788 (25.2) | 451 (11.7) |
|  | **CNN** | 1230 (81.8)** | 1507 (57.4)** | 929 (31.2)** | 500 (15.5)** |
|  | **SNQ** | 130 (26.1) | 68 (10.9) | 38 (5.3) | 16 (1.9) |
| **Mortality** | **ANZNN** | 377 (22.9) | 325 (12.2) | 167 (5.3) | 79 (2.1) |
|  | **CNN** | 478 (31.8)** | 282 (10.7) | 130 (4.4) | 55 (1.7) |
| **Chronic Lung Disease** | **SNQ** | 209 (41.9) | 199 (31.8) | 114 (16.0) | 64 (7.4) |
|  | **ANZNN** | 644 (39.1) | 829 (31.0) | 506 (16.2) | 254 (6.6) |
|  | **CNN** | 638 (42.4) | 986 (37.6)** | 573 (19.3)* | 275 (8.5)* |
| **Severe Neurological Injury** | **SNQ** | 88 (17.6) | 60 (9.6) | 35 (4.9) | 19 (2.2) |
|  | **ANZNN** | 263 (16.0) | 225 (8.4) | 118 (3.8) | 105 (2.7) |
|  | **CNN** | 310 (20.6)* | 353 (13.4)** | 241 (8.1)** | 140 (4.4)** |
| **Retinopathy of Prematurity** | **SNQ** | 77 (15.4) | 51 (8.2) | 12 (1.7) | 4 (0.5) |
|  | **ANZNN** | 209 (12.7) | 206 (7.7) | 53 (1.7) | 19 (0.5) |
|  | **CNN** | 298 (19.8)** | 239 (9.1) | 52 (1.8) | 7 (0.2) |
| **Necrotising Enterocolitis** | **SNQ** | 49 (9.8) | 46 (7.4) | 27 (3.8) | 12 (1.4) |
|  | **ANZNN** | 169 (10.3) | 215 (8.0) | 115 (3.7) | 67 (1.7) |
|  | **CNN** | 171 (11.4) | 207 (7.9) | 156 (5.2)* | 107 (3.3)** |
| **Nosocomial Infection** | **SNQ** | 175 (35.1) | 147 (23.5) | 84 (11.8) | 48 (5.6) |
|  | **ANZNN** | 539 (32.7) | 653 (24.4) | 426 (13.6) | 251 (6.5) |
|  | **CNN** | 557 (37.0)† | 761 (29.0)** | 471 (15.8)* | 280 (8.7)** |
| Notes: ** indicates p<0.001, * p<0.01, and †P<0.05 (for comparison among SNQ, ANZNN and CNN). The p-values were based on the Chi-square test. | | | | | |

**Table S3: Cross comparison of predictive power of very low birth weight (VLBW) and very low gestational age (VLGA) based models**

| **Total Cohort** | **VLBW Internal validation Cohort** | **VLGA Internal validation**  **Cohort** |
| --- | --- | --- |
|  | N=8111 | N=9727 |
| Model for mortality based on VLBW cohort | 0.830 | 0.843 |
| Model for mortality based on VLGA cohort | 0.829 | 0.843 |
| Model for CAO based on VLBW cohort | 0.834‡ | 0.830 |
| Model for CAO based on VLGA cohort | 0.832‡ | 0.830 |
| **For SNQ** | **VLBW Internal validation Cohort** | **VLGA Internal validation**  **Cohort** |
|  | N=900 | N=1142 |
| Model for mortality based on VLBW cohort | 0.810 | 0.863 |
| Model for mortality based on VLGA cohort | 0.810 | 0.864 |
| Model for CAO based on VLBW cohort | 0.844 | 0.850 |
| Model for CAO based on VLGA cohort | 0.844 | 0.850 |
| **For ANZNN** | **VLBW Internal validation Cohort** | **VLGA Internal validation**  **Cohort** |
|  | N=3769 | N=4529 |
| Model for mortality based on VLBW cohort | 0.826† | 0.825‡ |
| Model for mortality based on VLGA cohort | 0.831† | 0.831‡ |
| Model for CAO based on VLBW cohort | 0.847 | 0.839‡ |
| Model for CAO based on VLGA cohort | 0.846 | 0.841‡ |
| **For CNN** | **VLBW Internal validation Cohort** | **VLGA Internal validation**  **Cohort** |
|  | N=3442 | N=4056 |
| Model for mortality based on VLBW cohort | 0.842‡ | 0.856‡ |
| Model for mortality based on VLGA cohort | 0.834‡ | 0.849‡ |
| Model for CAO based on VLBW cohort | 0.813 | 0.809 |
| Model for CAO based on VLGA cohort | 0.812 | 0.808 |
| Notes: ‡:p<0.05; †: p<0.01; Two figures are significantly different if they share the same symbol. Chi square test was used for the comparison of predictive power. | | |

**Table S4: Comparisons of infant and perinatal characteristics and neonatal outcomes among networks (ANZNN, CNN, SNQ) for the 2 extreme components of the very low gestational age cohort and very low birth weight cohort 2008-2011 admissions**

|  |  |  | | | | | | | | | | | |
| --- | --- | --- | --- | --- | --- | --- | --- | --- | --- | --- | --- | --- | --- |
|  | | | | **VLBW Cohort**  **BW <1500 & GA ≥32 wk** | | | | | **VLGA Cohort**  **GA <32 & BW ≥1500** | | | | |
| **Networks** | | | ANZNN | | CNN | SNQ | Total | P-value | ANZNN | CNN | SNQ | Total | P-value |
| Total number (%) | | | 1368 | | 1130 | 262 | 2760 |  | 3686 | 3135 | 784 | 7605 |  |
| **Infant and Perinatal Characteristics** | | | | | |  |  |  |  |  |  |  |  |
| Gestational age, mean(SD) | | | 32.8(1.1) | | 32.9(1.14) | 32.9(1.1) | 32.8(1.1) | 0.07 | 30.5(0.75) | 30.4(0.79) | 30.5(0.7) | 30.4(0.76) | 0.008 |
| Birth weight, mean(SD) | | | 1301(172) | | 1330(140) | 1259(171) | 1309(161) | <0.0001 | 1741(200) | 1730(194) | 1729(200) | 1735(198) | 0.06 |
| Male sex | | | 632(46.3) | | 476 (42.2) | 112(42.8) | 1220(44.2) | 0.11 | 2233(60.6) | 1833(58.5) | 530(67.6) | 4596(60.4) | <0.0001 |
| Small for gestational age | | | 1120(81.9) | | 949(84.0) | 204(77.9) | 2273(82.4) | 0.053 | 303(8.2) | 345(11.0) | 65(8.3) | 713(9.3) | 0.0002 |
| Large for gestational age | | | 0 | | 0 | 0 | 0 |  | 634(17.2) | 539(17.2) | 127(16.2) | 1300(17.1) | 0.78 |
| **Neonatal Outcomes** | | | | |  |  |  |  |  |  |  |  |  |
| Mortality | | | 14(1.02) | | 17 (1.50) | 1 (0.38) | 32(1.16) | 0.25 | 10(1.3) | 59(1.6) | 59(1.9) | 128(1.68) | 0.43 |
| Composite adverse outcome | | | 46 (3.36) | | 82(7.26) | 3(1.15) | 131(4.74) | <0.0001 | 301(8.17) | 461(14.7) | 63(8.04) | 825(10.8) | <0.0001 |

N (%) are shown unless specified. Notes: the reported p-values were based on chi-square tests for categorical variables, and F tests for continuous variables. Composite adverse outcome is defined as: death or any major morbidities including chronic lung disease, severe neurological injury, necrotising enterocolitis, severe retinopathy.
